# Quercetin Alleviates the Progression of Breast Cancer-Related Depression via Inhibiting the Pyroptosis and Promoting the Immune Response

**DOI:** 10.1155/2022/8011988

**Published:** 2022-03-03

**Authors:** Qing Zhu, Lei Yang, Hui Yang, Yuanshan Han, Yun Chen, Ying He

**Affiliations:** ^1^Pharmacy Department of Hunan Cancer Hospital/the Affiliated Cancer Hospital of Xiangya School of Medicine, Central South University, Changsha, 410013 Hunan, China; ^2^Department of Pharmacy, The First Hospital of Hunan university of Chinese Medicine, Changsha, 410007 Hunan, China; ^3^The Second Department of Breast Surgery, Hunan Cancer Hospital/the Affiliated Cancer Hospital of Xiangya School of Medicine, Central South University, Changsha, 410013 Hunan, China

## Abstract

**Background:**

Breast cancer-related depression (BCRD) seriously inhibits the life quality of patients with breast cancer. The Xiaoyao Kangai Jieyu Formula is known to inhibit the progression of depression. However, the detailed function of the Xiaoyao Kangai Jieyu Formula in BCRD remains unclear.

**Methods:**

Network pharmacology was constructed to assess the downstream target of the Xiaoyao Kangai Jieyu Formula in BCRD. In addition, the tail suspension test, sucrose preference test, and forced swimming test were used to test the symptom of depression in mice. Fluoro-Jade B staining was performed to observe the structure of neurons. RT-qPCR and western blot were applied to evaluate mRNA and protein levels. Besides, ELISA was performed to test the inflammatory responses and the immune response-related cytokines.

**Results:**

Quercetin was identified as the key component of the Xiaoyao Kangai Jieyu Formula. Quercetin significantly inhibited BCRD-induced neuron pyroptosis via downregulation of PYD and card domain containing (ASC), NLR family pyrin domain containing 3 (NLRP3), and caspase-1, and quercetin could reverse BCRD-caused inhibition of neuron viability. Quercetin significantly attenuated the symptom of BCRD in mice, and it could reverse the contents of 5-hydroxytryptamine (5-HT), dopamine (DA), and neutrophil elastase (NE) in mice. Moreover, quercetin could promote the immune responses in xenograft mice via upregulation of interleukin- (IL-) 2, interferon-*γ* (IFN-*γ*), and IL-10.

**Conclusion:**

Quercetin, the active ingredient of the Xiaoyao Kangai Jieyu Formula, effectively mitigated the progression of BCRD by inhibiting pyroptosis, promoting immune response, and improving serum metabolism.

## 1. Introduction

Patients with breast cancer often exhibit significant emotional instability, especially depression, and the probability of being accompanied by depression has been reached at 40% [1, 2]. Meanwhile, depression is known to affect the life quality of patients seriously and even decrease the survival time of patients with breast cancer by 10%-20% [3]. Therefore, it is of great significance to study the pathogenesis of BCRD for the prevention and treatment of BCRD. At present, the main method of clinical treatment of BCRD is to give adjuvant antidepressant treatment after diagnosis [4]. However, the outcomes remain still limited as the mechanism by which breast cancer develops into BCRD remains largely unknown.

Traditional Chinese medicine is based on dialectical treatment, which has a significant therapeutic effect on the progression of breast cancer and drug resistance [5, 6]. Moreover, traditional Chinese medicine usually has unique advantages in the treatment of depression [7, 8]. The Xiaoyao Kangai Jieyu Formula is a traditional Chinese herbal compound that we have previously found to be highly effective in the treatment of BCRD [9]. Therefore, formulating prescriptions and formulating drugs (starting from traditional Chinese medicine) based on the etiology and pathogenesis of BCRD have a broader prospect and application value.

Previous studies suggested that the pathogenesis of BCRD mainly focused on autonomic neuroimmunity, neuroendocrine immunity, and oxidative stress injury, which are related to inflammation [10, 11]. In addition, cell pyroptosis is a novel form of cell death (programmed) discovered in recent years, and it is considered to be the most closely related form of cell damage to inflammation [12]. It has been documented that the classical cleavage pathway is mediated by aspartate-specific caspase-1 (aka caspase-1), which can be induced to be upregulated by inflammatory vesicles [13]. Our preliminary data revealed that inflammatory vesicles (NLRP3) are frequently activated during the progression of neurons in BCRD mice. Hence, it is hypothesized that the hippocampal neuron pyroptosis might participate in the occurrence and development of BCRD.

Based on the above background, our study was aimed at investigating the function of the Xiaoyao Kangai Jieyu Formula in inflammatory responses during the progression of BCRD. We hope that this work will exert novel insights into discovering the new methods against BCRD.

## 2. Material and Methods

### 2.1. Data Sources of Network Pharmacology

The active constituents of the Xiaoyao Kangai Jieyu Formula were obtained in 3 steps as previously described [14].

The target protein was predicted using the TCMSP database and DrugBank (https://www.drugbank.ca/) and TCMSP database. Other active components (the target proteins were not able to be predicted in these two databases) were analyzed by using SwissTargetPrediction, and the top targets predicted were selected according to the results. Each active constituent and the Xiaoyao Kangai Jieyu Formula active constituent-target protein network were coded by applying Cytoscape (v3.7.1) software for exploring the association between active constituents and target proteins. Additionally, Network Analyzer (a Cytoscape plugin) was applied to investigate the betweenness centrality of the network and the degree.

### 2.2. Potential Active Target Proteins (PATPs)

The intersection between the targets of the Xiaoyao Kangai Jieyu Formula and proteins (related to BCRD) was taken as the PATPs for the following analysis.

### 2.3. Protein-Protein Interaction (PPI) Investigation

In order to clarify the associations between PATPs, the STRING database was applied to establish the network of PPI. Moreover, the species were only adaptive to Homo sapiens, and a confidence score of 0.95 (<0.4 was considered low confidence, ≤0.7 regarded as medium, and >0.7 considered high) was selected in this analysis. Further experiments used the PPI data. Then, CytoNCA (a Cytoscape plugin) was applied to assess the network of PPI, and the network was established by top proteins (*n* = 150).

### 2.4. Gene Ontology (GO) Analysis

The molecular mechanisms of the Xiaoyao Kangai Jieyu Formula were determined using GO analysis. MCODE (a Cytoscape plugin) was applied in GO biological process analysis. In detail, the data of PPI were filtered in Cytoscape applying MCODE in this procedure. 0.05 was considered the significance level.

### 2.5. Kyoto Encyclopedia of Genes and Genomes (KEGG)

KEGG analysis was applied to explore potential pathways and biological functions in this work. In addition, KEGG analysis was applied with the ClusterProfiler package of the R language. 0.05 was considered the significance level.

### 2.6. Untargeted Metabolomics

Samples were placed on dry ice and weighed. A freeze tissue grinder was applied to homogenize the tissue. Tissue homogenate (200 *μ*L) was moved and mixed with 800 *μ*L of methanol/acetonitrile (1 : 1, *v*/*v*). The samples were vortexed for 30 s and centrifuged (14,500 rpm, 15 min, 4°C). The supernatant was evaporated to dryness by nitrogen blowing and then reconstituted with 100 *μ*L acetonitrile/water (1 : 1, *v*/*v*). Finally, the solution was filtered with a 0.22 *μ*m membrane and analyzed by UHPLC-QTOF-MS (Agilent 1290 UHPLC 6545 MS). The reaction conditions were as follows: a Waters HSS T3 column was applied to separate the sample solution (2 *μ*L) at 40°C with 0.1% formic acid (eluent A) and acetonitrile (eluent B) (the flow rate was 0.3 mL/min). A total 20-minute gradient program was set as follows: 0.01 min, 1% B; 1.5 min, 1% B; 13 min, 99% B; 16.5 min, 99% B; and equilibration time of 16.6 min at 1%.

### 2.7. Cell Culture

Breast cancer cells (4T1) were purchased from the Chinese Academy of Sciences. Nashed et al. showed that 4T1 facilitates the induction of depression-like states in mice [15]; therefore, 4T1 cells were selected for the study of breast cancer-related depression in this study. In addition, primary neurons originated from Invitrogen (item number: A15585, Waltham, MA, USA). Cells were maintained in DMEM (Invitrogen) with 10% FBS (Gibco) and penicillin (100 U/mL) in the condition of 37°C and 5% CO_2_.

### 2.8. Drug

The Xiaoyao Kangai Jieyu Formula was obtained from Changdu Zhenxing Lo. Ctd. (Hunan, China). The Xiaoyao Kangai Jieyu Formula consists of Bupleurum, Angelica, white peony, poria, Atractylodes, Prunella vulgaris, ginseng, turmeric, Hypericum perforatum, and roasted licorice.

### 2.9. Cell Treatment

4T1 cells were exposed to LPS (Sigma, 10 *μ*g/mL) for 6 h. Then, cells were centrifuged, and the supernatants were collected. The cell supernatants were added to primary neurons for 6 h. For the CORD group, primary neurons were exposed to 200 *μ*M CORT for 6 h. 4T1 cells were exposed to LPS (Sigma, 10 *μ*g/mL) for 6 h to simulate BCRD in vitro. Then, cells were centrifuged, and the supernatants were collected. The cell supernatants and 200 *μ*M CORT were added to primary neurons for 6 h [16].

### 2.10. In Vivo Model

Nude mice (BALB/c, *n* = 40; aged 6-8 weeks) originated from Beijing Vital River. The mice were placed in the condition of SPF. 4T1 cells (10^7^/mL) were injected into mice (subcutaneously). The tumor volume was measured weekly. Then, mice (except the control group) were injected with 30 mg/kg CORT suspension subcutaneously.

Mice in the BCRD+Pac group were injected intraperitoneally with paclitaxel liposomes (20 mg/kg) weekly. Mice in the BCRD+Flu group were administered with fluoxetine hydrochloride (7.8 mg/kg) every day. Mice in the quercetin group were injected intraperitoneally with quercetin at an equivalent daily dose. In addition, mice in the control, depression, BCRD, and breast cancer group were administered with the same amount of distilled water. After 3 weeks of treatment, each group of mice was analyzed according to multiple indicators [16].

The Institutional Animal Care and Use Committees of the Affiliated Cancer Hospital of Xiangya School of Medicine, Central South University, approved the protocol of this work (number 2021-056). The treatment of animals during the experiment conforms to the standards of “Guiding Opinions on Being Kind to Experimental Animals” issued by the Ministry of Science and Technology in 2006.

### 2.11. Sucrose Preference Test

Decreasing sucrose preference is considered homologous anhedonia, inability to experience pleasure, which mimics depression. The water bottles (*n* = 2) were put in each cage for the purpose of evaluating animal habit influence. Mice were deprived of water and fasted for one day before the experiments. Briefly, sucrose solution (2%) and the drinking water were put in a cage for 6 hours. Finally, measure the liquid content and use the following formula to calculate the sucrose preference as previously described [17].

### 2.12. Forced Swimming Test

The forced swimming test (FST) is used to measure changes in depressive behavior. The mice received FST, which was in line with the recent work [18]. Put the animal in a glass bottle with a height of 21 cm and a diameter of 16.5 cm. The bottle was added with water (13 cm deep) for 6 minutes. Force the mice to swim for 6 minutes, and observe how long they were motionless in the last 4 minutes. When heads of mice were out of the water and there was no significant movement of the limbs, the mice were defined as static. The result of the resting time was recorded.

### 2.13. Tail Suspension Test

The mice were individually suspended with tap and separated from each other. Then, the tape was placed 1 cm from the tail tip. The status of the mice was observed for 6 minutes, and the result was recorded.

### 2.14. CCK8 assay

Cells (5 × 10^3^ per well) were treated for 28 h. Then, cells were treated with CCK8 reagents (10 *μ*L, Beyotime) at 37°C for 2 h. After that, the absorbance (450 nm) was detected by using a microplate reader.

### 2.15. EthD-III staining

In brief, EthD-III (2 *μ*M) was applied to incubate the cells for 45 min and DAPI was applied to stain the cells. Confocal microscopy (LSM710, Carl Zeiss, Germany) was applied to visualize cells (522/593 nm).

### 2.16. RT-qPCR

TRIzol (Takara, Tokyo, Japan) was applied to isolate tissues or cells from total RNAs. The PrimeScript RT reagent Kit (Takara) was applied to synthesize cDNAs. The ABI7500 system was performed in RT-qPCR performing SYBR Green. RT-qPCR was applied as follows: 94°C for 2 min, followed by 35 cycles (94°C for 30 s and 55°C for 45 s). The primers originated from GenePharma (Shanghai, China). The 2^−△△CT^ method was applied in quantification. *β*-Actin was considered to be the reference control. The sequences are shown in Table 1.

### 2.17. Western blot

RIPA buffer was applied to isolate protein from tissues or cell lysates. BCA (Invitrogen) was applied in protein quantification. Subsequently, SDS-PAGE (10%) was applied to separate the proteins (40 *μ*g/lane), and the proteins were then transferred onto PVDF (Invitrogen). Primary antibodies were applied to incubate the membranes overnight after membranes were blocked with skim milk (3%) for 1 h. Afterward, a secondary antibody (anti-rabbit, Abcam; 1 : 5000) was applied to incubate the membranes for 1 h. The Odyssey Imaging System was applied to scan the membranes, and Odyssey v2.0 software (LICOR Biosciences, USA) was applied to analyze the blots. The primary antibodies were as follows: anti-NLRP3 (1 : 1000), anti-ASC (1 : 1000), anti-caspase-1 (1 : 1000), and anti-*β*-actin (1 : 1000). All antibodies originated from Abcam (USA). *β*-Actin was applied for quantification.

### 2.18. ELISA

The ELISA kit originated from Jiancheng, Nanjing (China). The levels of 5-HT (item number: H104-1-1), DA (item number: H170), NE (item number: H344-1), IL-2 (item number: H003), IFN-*γ* (item number: H025), and IL-10 (item number: H009-1) in the hippocampal/tumor homogenate of mice were determined.

### 2.19. Flow Cytometry

The tumors were dissociated and suspended. CD8-APC and CD4-FITC were subsequently applied to stain the cells for 15 minutes at 4°C. Afterward, cells were permeabilized for Foxp3-PE (eBiosciences) staining. Flow cytometry (BD FACSAria, USA) was applied to analyze the cells after cells. FlowJo (BD) was applied to quantify the data.

### 2.20. Histological Examination

KCl (10%) was applied to arrest the tissues, and a Canon camera (Tokyo) was applied to capture the images of tissues. Hematoxylin and eosin (H&E) were applied to stain the sections (4 *μ*m). The cross section was measured at the nucleus level in sectioned myocytes (longitudinally). A microscope (Olympus, Japan) was used to obtain the images. Image-Pro (Plus 6.0, NIH, USA) was applied to analyze the data.

### 2.21. Statistical Analysis

Data analysis was applied by using SPSS v18.0 (USA). Means ± SEM was applied to express the data. ANOVA (followed by the Tukey–Kramer post hoc test) was applied to compare the differences among experimental groups. *p* < 0.05 indicated significant changes.

## 3. Results

### 3.1. The Active Ingredient-BCRD-Target Network

**Figure 1:**
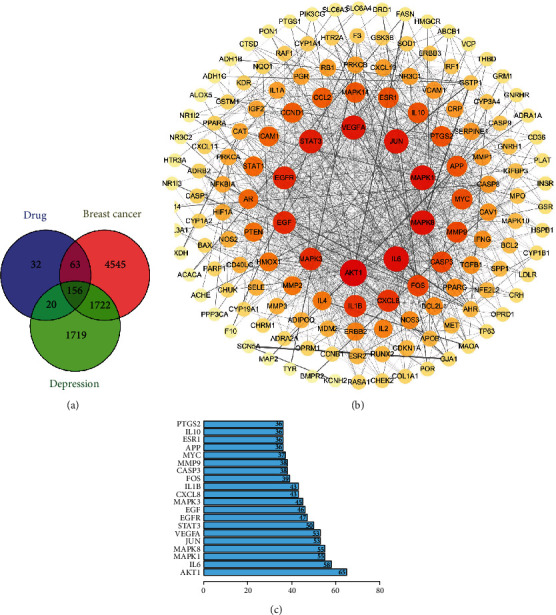
Active targets and PPI network. (a) The overlapped potential active target proteins were presented. (b) The network of PPI containing the active targets was presented. The redder the color, the greater the degree value. The line from thick to thin indicates that the edge betweenness is from big to small. (c) Prediction of the top 20 key targets. The horizontal coordinate is the degree value of each target.

A total of 156 targets were obtained through a comprehensive analysis of the active ingredients of the Xiaoyao Kangai Jieyu Formula, breast cancer, and depression (Figure 1(a)). We constructed PPI networks for these 156 target genes (Figure 1(b)). Genes with a degree score greater than the average score were selected as key targets, and a total of 53 key targets were screened out. The top 20 targets are shown in Figure 1(c).

Core genes were screened by MCODE analysis. Four gene clusters and four core genes were obtained: APP, AKT1, IL10 and POR, respectively (Table 2).

Based on the included active ingredients, BCRD, and targets, the active ingredient-disease-target network was constructed (Supplementary Figure 1).

### 3.2. GO and KEGG Enrichment Analysis

**Figure 2:**
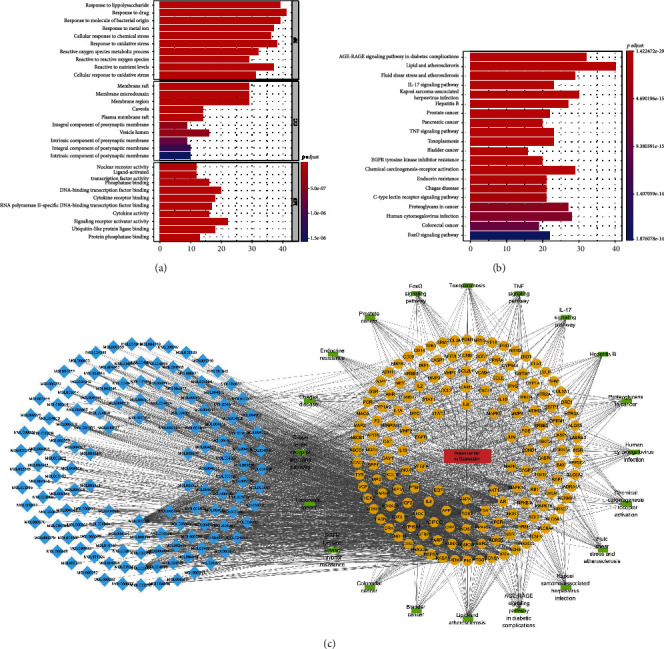
The GO data of enrichment and KEGG analysis. (a) GO analysis was performed to investigate the cellular biological process. (b) KEGG analysis was performed to investigate the most enriched pathways. (c) The network of constituents-diseases-pathways-targets.

Next, GO analysis showed that the active components of the compound were mainly involved in response to lipopolysaccharide, response to drug, and response to molecule of bacterial origin (Figure 2(a)). In addition, KEGG analysis showed that the active components of the compound were related to the following pathways: AGE-RAGE signaling pathway in diabetic complication, lipid and atherosclerosis, and fluid shear stress and atherosclerosis (Figure 2(b)). We constructed a PPI network based on the potential targets and pathways of the Xiaoyao Kangai Jieyu Formula's active components in BCRD (Figure 2(c)). Furthermore, according to the degree of the active ingredient, we found that quercetin is the most important ingredient in the Xiaoyao Kangai Jieyu Formula (Supplementary Table 2).

### 3.3. The Occurrence of BCRD Induced the Pyroptosis of Neurons

**Figure 3:**
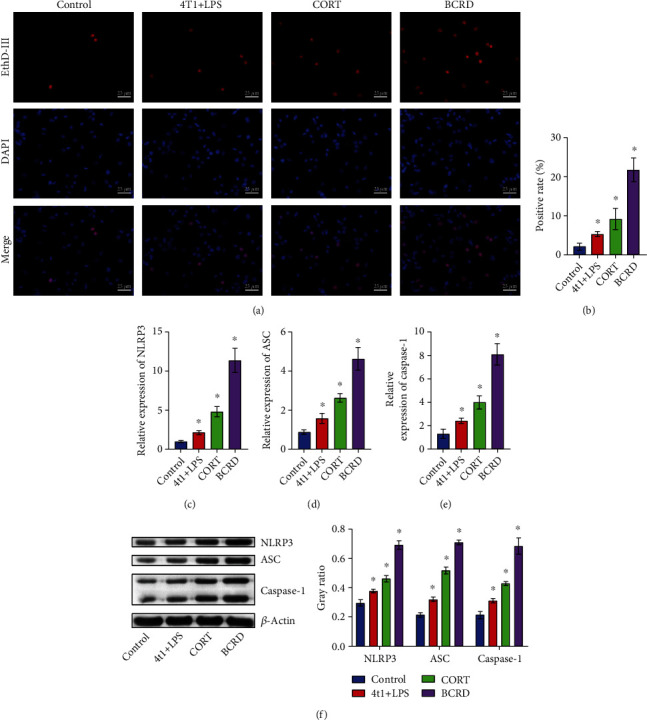
The occurrence of BCRD induced the pyroptosis of neurons. Neurons were treated with LPS+4T1, LPS+4T1+BCRD, or LPS+4T1+CORD. (a, b) EthD-III staining was used to observe the membrane pores of neurons. (c–e) Caspase-1, ASC, and NLRP3 levels in neurons were assessed by RT-qPCR. (f) ASC, caspase-1, and NLRP3 levels in neurons were investigated by western blot. *β*-Actin was applied for quantification. ^∗^*p* < 0.05 compared with control.

To mimic BCRD *in vitro*, neurons were exposed to the supernatants of LPS-treated 4T1 cells. In Figures 3(a) and 3(b), the number of pyroptotic cells was markedly elevated in BCRD. Meanwhile, the levels of ASC, NLRP3, and caspase-1 in neurons were obviously upregulated in BCRD (Figures 3(c)–3(f)).

### 3.4. Quercetin Significantly Reversed BCRD-Induced Cell Pyroptosis

**Figure 4:**
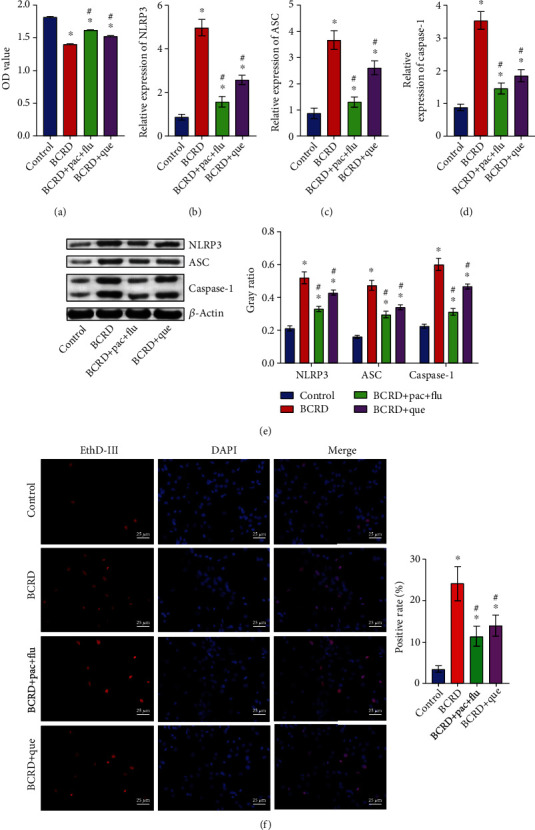
Quercetin significantly reversed BCRD-induced cell pyroptosis. Neurons were divided into BCRD, BCRD+quercetin, or BCRD+Pac+Flu. (a) The viability of neurons was assessed by the CCK8 assay. (b–d) The levels of NLRP3, ASC, and caspase-1 in neurons were investigated by RT-qPCR. (e) ASC, NLRP3, and caspase-1 levels in neurons were investigated by western blot. *β*-Actin was applied for quantification. (f) The pyroptosis of hippocampal neurons was evaluated by EthD-III staining ^∗^*p* < 0.05 compared with control. ^#^*p* < 0.05 compared with BCRD.

Since quercetin was identified as the key constituent of the Xiaoyao Kangai Jieyu Formula, the following analysis was applied to analyze the impact of quercetin on BCRD progression. The data confirmed that the viability of neurons was significantly decreased by the supernatants of LPS-treated 4T1 cells, which was significantly reversed by Pac+Flu or quercetin (Figure 4(a)). Consistently, the supernatants of LPS-treated 4T1 cells markedly upregulated ASC, NLRP3, and caspase-1 levels in neurons, while this phenomenon was partially restored by Pac+Flu or quercetin (Figures 4(b)–4(e)). Meanwhile, Pac+Flu or quercetin obviously reversed the effect of supernatants of LPS-treated 4T1 cells on cell pyroptosis (Figure 4(f)).

### 3.5. Quercetin Alleviated Neuron Injury and Depressive Behavior in BCRD Mice

**Figure 5:**
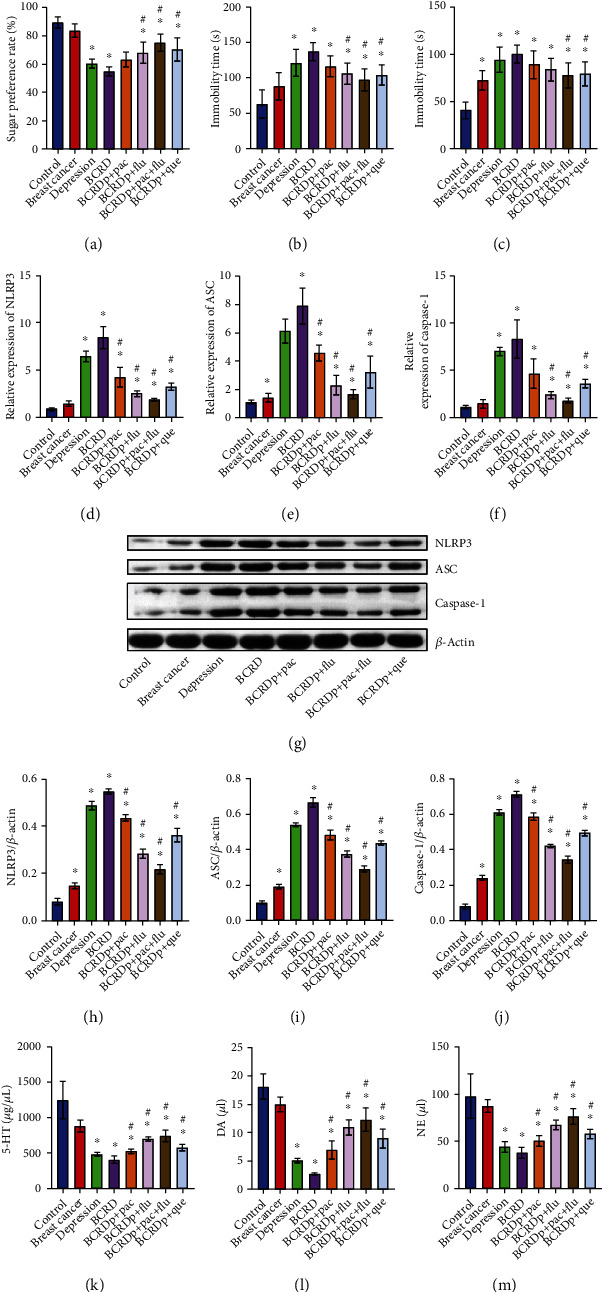
Quercetin alleviated neuron injury and depressive behavior in BCRD mice. (a) The sugar preference rate of mice was detected. (b) The immobility time was recorded after the forced swimming test. (c) The immobility time was recorded after the tail suspension test. (d–f) ASC, NLRP3, and caspase-1 levels in neurons were assessed by RT-qPCR. (g–j) NLRP3, ASC, and caspase-1 levels in neurons were investigated by western blot. *β*-Actin was applied for quantification. (k–m) The contents of 5-HT, DA, and NE in mice were detected by ELISA. ^∗^*p* < 0.05 compared with control. ^#^*p* < 0.05 compared with BCRD (*n* = 6).

To assess the function of quercetin in BCRD *in vivo*, an *in vivo* model of BCRD was constructed. As demonstrated in Figures 5(a)–5(c), the symptom of depression in mice was significantly decreased in BCRD and depression, which was markedly reversed by quercetin or Pac+Flu. In addition, BCRD and depression notably upregulated ASC, NLRP3, and caspase-1 levels in mice, while quercetin or Pac+Flu partially rescued this phenomenon (Figures 5(d)–5(j)). Furthermore, the contents of 5-HT, DA, and NE in mice were much higher in the BCRD and depression group, which was rescued in the presence of quercetin or Pac+Flu (Figures 5(k)–5(m)).

### 3.6. Quercetin Promoted an Antitumor Immune Response in BCRD Mice

**Figure 6:**
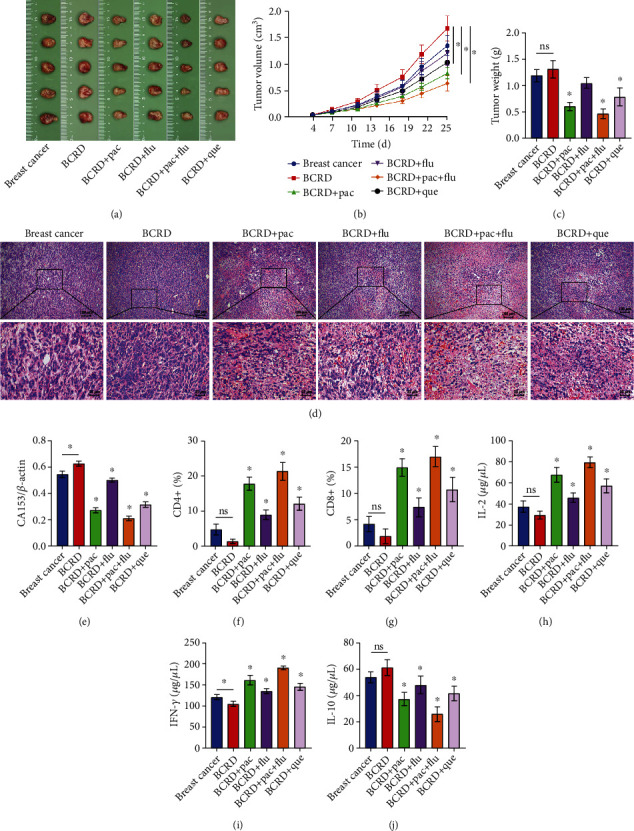
Quercetin promoted an anti-tumor immune response in BCRD mice. (a) The tumor tissues of mice were collected. (b) The tumor volume was calculated. (c) The tumor weight was recorded. (d) The histological change in mice was detected by H&E staining. (e) The expression of CA153 was assessed. (f, g) The ratio of CD4+ and CD8+ in mice was assessed by flow cytometry. (h–j) IL-2, IFN-*γ*, and IL-10 levels in mice were investigated by ELISA. ^∗^*p* < 0.05 compared with breast cancer (*n* = 6).

A xenograft mouse model was constructed to assess the effect of quercetin on immune response in BCRD mice. The result indicated that BCRD further increased the tumor sizes and weight in breast cancer mice, while this phenomenon was notably revised by quercetin or Pac+Flu (Figures 6(a)–6(c)). In addition, BCRD significantly induced the injury in tissues, and the effect of BCRD was significantly inhibited in the presence of quercetin or Pac+Flu (Figure 6(d)). Meanwhile, the content of CA153 in BCRD mice was markedly inhibited by Pac+Flu or quercetin (Figure 6(e)), and BCRD-induced decrease in CD4+ and CD8+ was also restored by quercetin or Pac+Flu (Supplementary Figure 2, Figures 6(f) and 6(g)). Furthermore, BCRD greatly reduced IFN-*γ* and IL-2 levels and increased IL-10 in mice, which was notably rescued by quercetin or Pac+Flu (Figures 6(h)–6(j)).

### 3.7. Quercetin Partially Reversed Serum Metabolism in BCRD Mice

**Figure 7:**
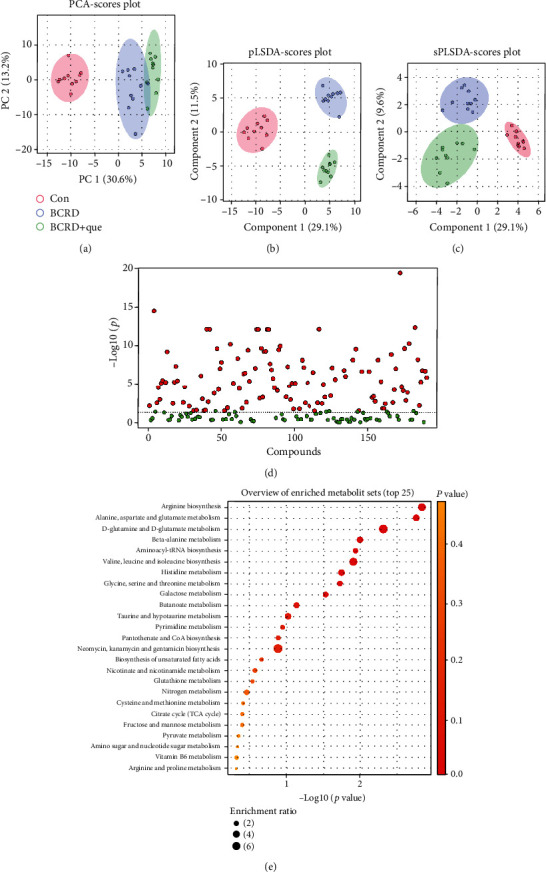
Quercetin might promote the antitumor immune response in BCRD mice via regulation of serum metabolism. (a) Principal component analysis. (b) Partial least squares discriminant analysis (PLS-DA). (c) Sparse partial least squares discriminant analysis (SPLS-DA). (d) Differential metabolites are represented by a volcano plot. (e) KEGG pathway enrichment analysis of differential metabolites.

As revealed in Figures 7(a)–7(c), the metabolites in different experimental groups were divided into different clusters. The volcano plot showed that 115 metabolites varied significantly among the three groups (Figure 7(d)). We noticed that quercetin effectively reversed the levels of certain metabolites in BCRD, such as argininosuccinic acid, L-asparagine, gamma-aminobutyric acid, succinic acid, and uracil (Supplementary Figure 3). KEGG analysis showed that these differential metabolites were mainly enriched in arginine biosynthesis, alanine, aspartate and glutamate metabolism, and D-glutamine and D-glutamate metabolism (Figure 7(e)).

## 4. Discussion

BCRD seriously impairs the quality of life of patients. It was reported that traditional Chinese medicine could inhibit cancer and depression development [7, 19]. However, the function of traditional Chinese medicine in BCRD needs to be further analyzed. In this research, the Xiaoyao Kangai Jieyu Formula could inhibit the progression of BCRD. In addition, quercetin was identified as the key active constituent of the Xiaoyao Kangai Jieyu Formula. Additionally, this work found the association between quercetin and serum metabolism in BCRD. Thus, we explored the function of quercetin, the active ingredient of Xiaoyao Kangai Jieyu Formula, in BCRD. Our findings suggest that quercetin may be used as a new agent for BCRD treatment.

This study assessed the mechanisms of the Xiaoyao Kangai Jieyu Formula on BCRD, and downstream proteins and the active constituents were predicted. The Xiaoyao Kangai Jieyu Formula active constituent-target network revealed the pharmacological foundation of the Xiaoyao Kangai Jieyu Formula. The targets and active constituents were hypothesized in this section. Flavonoids are the main active constituents of the Xiaoyao Kangai Jieyu Formula. Moreover, quercetin is known to be a polyphenolic flavonoid that has antitumor activity, and it exerts in the source of vegetal food and multiple traditional Chinese medicines [20, 21]. In vivo and in vitro study accumulation has paid attention to the chemopreventive activity and mechanisms underlying the function of quercetin BCRD.

Meanwhile, quercetin has multiple biological activities. For example, quercetin attenuates LPS-induced depression-like behavior and learning memory impairment in rats, which may be related to its modulation in the imbalance of hippocampal Copine 6 and TREM1/2 expression associated with brain-derived neurotrophic factor [22]. Quercetin could inhibit the behavior of chronic unpredictable mild stress-induced depression through involving nuclear factor-E2-related factor 2 [23]. Numerous studies have indicated that quercetin plays a therapeutic role in a variety of cancers. For example, quercetin inhibits cervical cancer cell invasion by reducing UBE2S expression in cervical cancer [24]. Quercetin targets specific signaling pathways, which is considered a promising component for treating glioblastoma multiforme [25]. Besides, quercetin has also been shown to have positive anticancer effects in ovarian cancer [26]. These studies suggest that quercetin is a prospective candidate in the treatment of both depression and cancer.

Recent studies indicated that pyroptosis plays a crucial role in the cellular process [27, 28]. Caspase-1, NLRP3, and ASC are known to be the important modulators in cell pyroptosis [29, 30]. Meanwhile, IL-1*β* is also a pyroptosis-related cytokine [31, 32]. Caspase-1 was often upregulated during the onset of neuronal injury, suggesting that the promotion of pyroptosis might be associated with neuronal injury [33, 34]. Our study revealed that quercetin could reverse the neuron pyroptosis via upregulating cleaved caspase-1, NLRP3, and ASC. Isoglycyrrhizin, a flavonoid similar to quercetin, has recently been demonstrated to improve depression by inhibiting NLRP3-mediated cellular pyroptosis via the miRNA-27a/SYK/NF-*κ*B axis [35]. In this work, we firstly found the correlation between quercetin and pyroptosis in BCRD, suggesting that quercetin reversed the neuron injury during the progression of BCRD through promoting pyroptosis.

It has been reported that disruption to metabolism could lead to the progression of depression [36]. This study found that argininosuccinic acid, L-asparagine, gamma-aminobutyric acid, succinic acid, and uracil could be inhibited by quercetin in BCRD mice. L-Asparagine was known to be the key marker in cardiovascular diseases [37]. An excessive increase in gamma-aminobutyric acid could cause metabolic disorders, which further resulted in the development of diabetes [38]. Succinic acid played a key role in the chronic diseases of the elder people [39], and uracil was the key marker in the metabolic cycle [40]. Thus, our finding revealed that the progression of BCRD might be closely correlated with the metabolic disorder, and quercetin has been shown to partially regulate the abnormalities of relevant metabolites in BCRD.

There are some limitations in this work as follows: (1) the mechanisms by which quercetin regulates the progression of BCRD remain unclear and (2) the relation between quercetin and serum metabolism-related signaling is needed to be analyzed. Hence, more analysis is essential in the future.

## 5. Conclusions

To sum up, quercetin, the active ingredient of the Xiaoyao Kangai Jieyu Formula, effectively mitigated the progression of BCRD by inhibiting pyroptosis, promoting immune response, and improving serum metabolism.

## Figures and Tables

**Table 1:** 

Gene	Primer sequence	Gene ID	Length
NLRP3	FCCTCTTTGGCCTTGTAAACCAG RTGGCTTTCACTTCAATCCACT	216799	113 bp
ASC(PYCARD)	FCAGAGTACAGCCAGAACAGGACACT RAAGCATCCAGCACTCCGTCCAC	66824	91 bp
Caspase-1	FACAAGGCACGGGACCTATG RTCCCAGTCAGTCCTGGAAATG	12362	237 bp
*β*-Actin	FACATCCGTAAAGACCTCTATGCC RTACTCCTGCTTGCTGATCCAC	1146	223 bp

**Table 2:** Cluster analysis based on Molecular Complex Detection.

Cluster	Network	Nodes	Edges	Node IDs
1	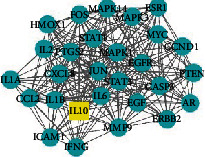	28	254	FOS, STAT1, CXCL8, STAT3, ICAM1, IL1A, IL1B, MYC, AR, CCL2, HMOX1, ERBB2, IFNG, MAPK3, EGF, MMP9, PTEN, JUN, MAPK14, CCND1, IL2, MAPK1, EGFR, IL10, ESR1, IL6, CASP3, PTGS2
2	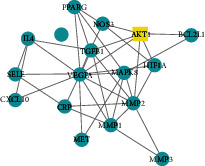	16	39	SELE, MET, PPARG, BCL2L1, CXCL10, IL4, NOS3, VEGFA, CRP, TGFB1, HIF1A, MAPK8, MMP2, MMP1, MMP3, AKT1
3	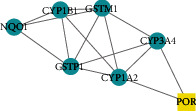	7	14	GSTP1, CYP3A4, CYP1B1, CYP1A2, NQO1, POR, GSTM1
4	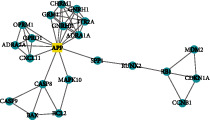	22	47	RUNX2, APP, CXCL11, OPRD1, BAX, ADRA2A, HTR2A, GRM1, CHRM1, RB1, CASP8, GNRH1, CDKN1A, MAPK10, GNRHR, BCL2, ADRA1A, CASP9, SPP1, MDM2, CCNB1, OPRM1

## Data Availability

The data used to support the findings of this study are available from the corresponding author upon request.

## Supplementary Materials

Supplementary 1 Supplementary Table 2: the information of key ingredients.

Supplementary 2 Supplementary Figure 1: the network of active ingredient-disease-target.

Supplementary 3 Supplementary Figure 2: CD4+ and CD8+ ratios in mice.

Supplementary 4 Supplementary Figure 3: the heat map of differential metabolites in plasma of mice.

Supplementary 5 Supplementary Figure 4: primary mouse neuronal cell identification. (A) Representative image of primary mouse cortical neurons. (B) IF for identification of primary neurons.
