# Supplementary material for: Quercetin Alleviates the Progression of Breast Cancer-Related Depression via Inhibiting the Pyroptosis and Promoting the Immune Response
Source: Mediators Inflamm. 2022 Mar 3;2022:8011988. doi: 10.1155/2022/8011988 (PMC8966747; doi:10.1155/2022/8011988)
Supplement: Supplementary 1 — Supplementary Table 2: the information of key ingredients. [file 8011988.f1.pdf]

Table 2. The information of key ingredients

MOL ID	Name	Average Shortest Path Length	Betweenness Centrality	Closeness Centrality	Degree
MOL000098	quercetin	1.979798	0.130149	0.505102	100
MOL000006	luteolin	2.410774	0.021713	0.414804	38
MOL000422	kaempferol	2.3367	0.030765	0.427954	37
MOL000358	beta-sitosterol	2.451178	0.014165	0.407967	24
MOL004328	naringenin	2.545455	0.011223	0.392857	22
MOL000392	formononetin	2.538721	0.003915	0.393899	19
MOL000497	licochalcone a	2.531987	0.00575	0.394947	19
MOL003896	7-Methoxy-2-methyl isoflavone	2.538721	0.003108	0.393899	19
MOL002565	Medicarpin	2.552189	0.004666	0.391821	18
MOL004978	2-[(3R)-8,8-dimethyl-3,4-dihydro-2H-pyrano[6,5-f]chromen-3-yl]-5-methoxyphenol	2.538721	0.002232	0.393899	18
MOL00	Vestitol	2.552189	0.002592	0.391821	17

0500					
MOL000791	bicuculline	2.558923	0.007969	0.390789	17
MOL004891	shinpterocarpin	2.552189	0.003206	0.391821	17
MOL004959	1-Methoxyphaseollidin	2.558923	0.001332	0.390789	16
MOL005003	Licoagrocarpin	2.552189	0.001357	0.391821	16
MOL000449	Stigmasterol	2.478114	0.013133	0.403533	15
MOL000787	Fumarine	2.585859	0.002985	0.386719	15
MOL004957	HMO	2.565657	0.00134	0.389764	15
MOL004966	3'-Hydroxy-4'-O-Methylglabridin	2.565657	0.001284	0.389764	15
MOL004974	3'-Methoxyglabridin	2.565657	0.001221	0.389764	15
MOL004835	Glypallichalcone	2.572391	0.001208	0.388743	14
MOL005007	Glyasperins M	2.579125	0.001144	0.387728	14
MOL000354	isorhamnetin	2.592593	7.30E-04	0.385714	13
MOL004811	Glyasperin C	2.579125	5.99E-04	0.387728	13
MOL004828	Glepidotin A	2.579125	7.62E-04	0.387728	13

MOL004833	Phaseolinisoflavan	2.572391	8.08E-04	0.388743	13
MOL004912	Glabrone	2.579125	5.53E-04	0.387728	13
MOL004991	7-Acetoxy-2-methylisoflavone	2.585859	7.06E-04	0.386719	13
MOL004824	(2S)-6-(2,4-dihydroxyphenyl)-2-(2-hydroxypropan-2-yl)-4-methoxy-2,3-dihydrofuro[3,2-g]chromen-7-one	2.592593	5.07E-04	0.385714	12
MOL004849	3-(2,4-dihydroxyphenyl)-8-(1,1-dimethylprop-2-enyl)-7-hydroxy-5-methoxy-coumarin	2.592593	5.39E-04	0.385714	12
MOL004908	Glabridin	2.585859	6.65E-04	0.386719	12
MOL004911	Glabrene	2.585859	4.75E-04	0.386719	12
MOL000417	Calycosin	2.599327	3.69E-04	0.384715	11
MOL000737	morin	2.606061	0.00326	0.383721	11
MOL003656	Lupiwighteone	2.592593	3.48E-04	0.385714	11
MOL004808	glyasperin B	2.599327	4.45E-04	0.384715	11

MOL004810	glyasperin F	2.599327	3.52E-04	0.384715	11
MOL004815	(E)-1-(2,4-dihydroxyphenyl)-3-(2,2-dimethylchromen-6-yl)prop-2-en-1-one	2.599327	3.52E-04	0.384715	11
MOL004820	kanzonols W	2.599327	3.52E-04	0.384715	11
MOL004856	Gancaonin A	2.592593	3.92E-04	0.385714	11
MOL004857	Gancaonin B	2.592593	4.78E-04	0.385714	11
MOL004864	5,7-dihydroxy-3-(4-methoxyphenyl)-8-(3-methylbut-2-enyl)chromone	2.599327	3.94E-04	0.384715	11
MOL004885	licoisoflavanone	2.599327	3.98E-04	0.384715	11
MOL004907	Glyzaglabrin	2.606061	4.27E-04	0.383721	11
MOL004915	Eurycarpin A	2.592593	3.48E-04	0.385714	11
MOL004961	Quercetin der.	2.599327	3.36E-04	0.384715	11
MOL005000	Gancaonin G	2.599327	4.44E-04	0.384715	11
MOL005012	Licoagroisoflavone	2.592593	3.48E-04	0.385714	11

MOL005016	Odoratin	2.599327	3.36E-04	0.384715	11
MOL004805	(2S)-2-[4-hydroxy-3-(3-methylbut-2-enyl)phenyl]-8,8-dimethyl-2,3-dihydropyrano[2,3-f]chromen-4-one	2.612795	3.13E-04	0.382732	10
MOL004841	Licochalcone B	2.606061	2.93E-04	0.383721	10
MOL004848	licochalcone G	2.612795	3.21E-04	0.382732	10
MOL004879	Glycyrin	2.606061	4.15E-04	0.383721	10
MOL004990	7,2',4'-trihydroxy－5-methoxy-3－arylcoumarin	2.612795	2.55E-04	0.382732	10
MOL005020	dehydroglyasperins C	2.599327	3.34E-04	0.384715	10
MOL000049	3β-acetoxyatractylone	2.612795	8.12E-04	0.382732	9
MOL000296	hederagenin	2.552189	0.005173	0.391821	9
MOL004827	Semilicoisoflavone B	2.612795	2.69E-04	0.382732	9
MOL004883	Licoisoflavone	2.612795	3.04E-04	0.382732	9
MOL004884	Licoisoflavone B	2.619529	2.36E-04	0.381748	9

MOL004980	Inflacoumarin A	2.606061	3.27E-04	0.383721	9
MOL005008	Glycyrrhiza flavonol A	2.612795	2.67E-04	0.382732	9
MOL005321	Frutinone A	2.612795	3.88E-04	0.382732	9
MOL001484	Inermine	2.639731	7.44E-04	0.378827	8
MOL003370	Onjixanthone I	2.632997	4.67E-04	0.379795	8
MOL004814	Isotrifoliol	2.632997	2.38E-04	0.379795	8
MOL004855	Licoricone	2.626263	2.82E-04	0.380769	8
MOL004863	3-(3,4-dihydroxyphenyl)-5,7-dihydroxy-8-(3-methylbut-2-enyl)chromone	2.626263	1.62E-04	0.380769	8
MOL004904	licopyranocoumarin	2.626263	2.64E-04	0.380769	8
MOL004945	(2S)-7-hydroxy-2-(4-hydroxyphenyl)-8-(3-methylbut-2-enyl)chroman-4-one	2.619529	2.34E-04	0.381748	8
MOL000239	Jaranol	2.626263	1.58E-04	0.380769	7
MOL002311	Glycyrol	2.646465	1.59E-04	0.377863	7

MOL003648	Inermin	2.653199	5.29E-04	0.376904	7
MOL004609	Areapillin	2.619529	1.69E-04	0.381748	7
MOL004806	euchrenone	2.632997	1.88E-04	0.379795	7
MOL004866	2-(3,4-dihydroxyphenyl)-5,7-dihydroxy-6-(3-methylbut-2-enyl)chromone	2.619529	2.06E-04	0.381748	7
MOL004910	Glabranin	2.632997	2.48E-04	0.379795	7
MOL004949	Isolicoflavonol	2.632997	1.27E-04	0.379795	7
MOL005017	Phaseol	2.639731	1.70E-04	0.378827	7
MOL005344	ginsenoside rh2	2.666667	0.001238	0.375	7
MOL005384	suchilactone	2.646465	3.86E-04	0.377863	7
MOL000490	petunidin	2.653199	8.98E-05	0.376904	6
MOL000522	arctiin	2.653199	2.05E-04	0.376904	6
MOL001792	DFV	2.639731	2.51E-04	0.378827	6
MOL002844	Pinocembrin	2.639731	2.51E-04	0.378827	6

MOL003330	(-)-Phillygenin	2.646465	2.60E-04	0.377863	6
MOL004598	3,5,6,7-tetramethoxy-2-(3,4,5-trimethoxyphenyl)chromone	2.639731	1.35E-04	0.378827	6
MOL004829	Glepidotin B	2.639731	2.04E-04	0.378827	6
MOL004898	(E)-3-[3,4-dihydroxy-5-(3-methylbut-2-enyl)phenyl]-1-(2,4-dihydroxyphenyl)prop-2-en-1-one	2.646465	8.07E-05	0.377863	6
MOL004941	(2R)-7-hydroxy-2-(4-hydroxyphenyl)chroman-4-one	2.639731	2.51E-04	0.378827	6
MOL004948	Isoglycyrol	2.639731	9.81E-05	0.378827	6
MOL005308	Aposiopolamine	2.936027	2.52E-04	0.340596	6
MOL003322	FORSYTHINOL	2.659933	1.23E-04	0.375949	5
MOL004798	delphinidin	2.659933	1.19E-04	0.375949	5
MOL004913	1,3-dihydroxy-9-methoxy-6-benzofurano[3,2-c]chromenone	2.835017	2.48E-05	0.352732	5
MOL00	1,3-dihydroxy-8,9-	2.814815	2.76E-05	0.355263	5

4914	dimethoxy-6-benzofurano[3,2-c]chromenone				
MOL004988	Kanzonol F	2.646465	7.90E-05	0.377863	5
MOL004989	6-prenylated eriodictyol	2.646465	7.86E-05	0.377863	5
MOL005001	Gancaonin H	2.646465	1.23E-04	0.377863	5
MOL005018	Xambioona	2.653199	6.59E-05	0.376904	5
MOL005356	Girinimbin	2.666667	1.17E-04	0.375	5
MOL000492	(+)-catechin	2.666667	4.64E-04	0.375	4
MOL000493	campesterol	2.606061	0.002357	0.383721	4
MOL003347	hyperforin	3.420875	4.16E-05	0.292323	4
MOL004653	(+)-Anomalin	2.673401	8.38E-05	0.374055	4
MOL004838	8-(6-hydroxy-2-benzofuranyl)-2,2-dimethyl-5-chromenol	2.666667	7.98E-05	0.375	4
MOL004882	Licocoumarone	2.814815	1.86E-05	0.355263	4
MOL004903	liquiritin	2.680135	3.34E-04	0.373116	4
MOL00	Sigmoidin-B	2.659933	8.24E-05	0.375949	4

4935					
MOL00 4993	8-prenylated eriodictyol	2.653199	5.73E-05	0.376904	4
MOL01 3187	Cubebin	2.666667	7.38E-05	0.375	4
MOL00 0359	sitosterol	3.313131	1.71E-04	0.301829	2
MOL00 0953	CLR	3.313131	1.71E-04	0.301829	2
MOL00 1645	Linoleyl acetate	2.700337	6.26E-06	0.370324	2
MOL00 1919	(3S,5R,8R,9R,10S,14S)-3,17-dihydroxy-4,4,8,10,14-pentamethyl-2,3,5,6,7,9-hexahydro-1H-cyclopenta[a]phenanthrene-15,16-dione	3.313131	1.71E-04	0.301829	2
MOL00 1924	paeoniflorin	3.441077	1.22E-05	0.290607	2
MOL00 2879	Diop	3.003367	4.50E-06	0.33296	2
MOL00 4355	Spinasterol	3.313131	1.71E-04	0.301829	2
MOL00 4624	Longikaurin A	3.313131	1.84E-05	0.301829	2
MOL00 4718	α -spinasterol	3.313131	1.71E-04	0.301829	2

MOL004924	(-)-Medicocarpin	2.700337	1.89E-05	0.370324	2
MOL005317	Deoxyharringtonine	2.888889	9.56E-04	0.346154	2
MOL005318	Dianthramine	2.700337	6.26E-06	0.370324	2
MOL005320	arachidonate	2.700337	6.26E-06	0.370324	2
MOL006767	Vulgaxanthin-I	2.700337	5.70E-06	0.370324	2
MOL000022	14-acetyl-12-senecioyl-2E,8Z,10E-atractylentriol	2.707071	0	0.369403	1
MOL000033	(3S,8S,9S,10R,13R,14S,17R)-10,13-dimethyl-17-[(2R,5S)-5-propan-2-yloctan-2-yl]-2,3,4,7,8,9,11,12,14,15,16,17-dodecahydro-1H-cyclopenta[a]phenanthren-3-ol	3.353535	0	0.298193	1
MOL000072	8β-ethoxy atractylenolide Ⅲ	2.707071	0	0.369403	1
MOL000211	Mairin	3.353535	0	0.298193	1
MOL000273	(2R)-2-[(3S,5R,10S,13R,14R,	3.393939	0	0.294643	1

	16R,17R)-3,16-dihydroxy-4,4,10,13,14-pentamethyl-2,3,5,6,12,15,16,17-octahydro-1H-cyclopenta[a]phenanthren-17-yl]-6-methylhept-5-enoic acid				
MOL000275	trametenolic acid	3.393939	0	0.294643	1
MOL000279	Cerevisterol	3.393939	0	0.294643	1
MOL000282	ergosta-7,22E-dien-3beta-ol	3.353535	0	0.298193	1
MOL000283	Ergosterol peroxide	3.353535	0	0.298193	1
MOL002776	Baicalin	3.043771	0	0.32854	1
MOL003315	3beta-Acetyl-20,25-epoxydammarane-24alpha-ol	3.454545	0	0.289474	1
MOL005348	Ginsenoside-Rh4_qt	3.393939	0	0.294643	1
MOL005376	Panaxadiol	3.454545	0	0.289474	1
MOL005399	alexandrin_qt	3.353535	0	0.298193	1

MOL006772	poriferasterol monoglucoside_qt	3.353535	0	0.298193	1
MOL006774	stigmast-7-enol	3.353535	0	0.298193	1
