# Supplementary figures and images for: Quercetin Alleviates the Progression of Breast Cancer-Related Depression via Inhibiting the Pyroptosis and Promoting the Immune Response
Source: Mediators Inflamm. 2022 Mar 3;2022:8011988. doi: 10.1155/2022/8011988 (PMC8966747; doi:10.1155/2022/8011988)

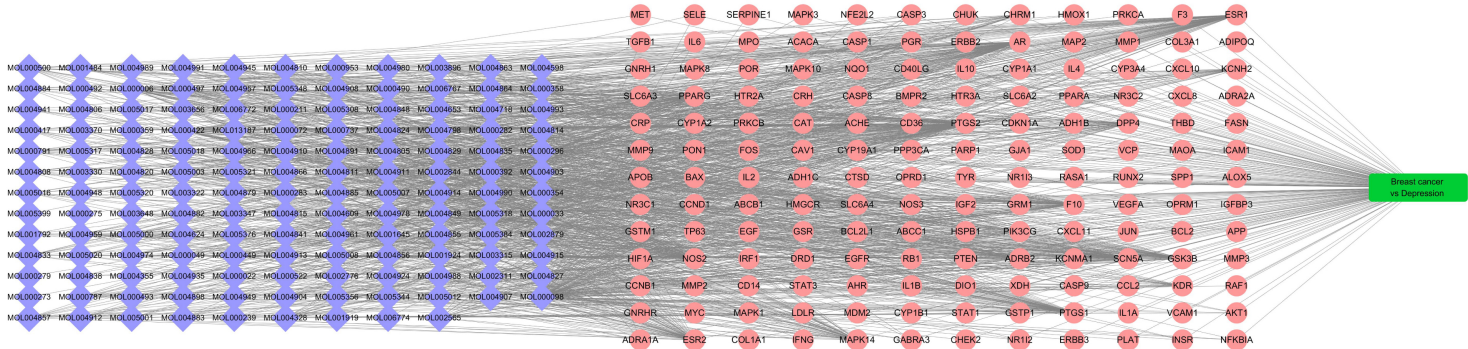

Supplement: Supplementary 2 — Supplementary Figure 1: the network of active ingredient-disease-target. [file 8011988.f2.pdf]

**A****CD4+**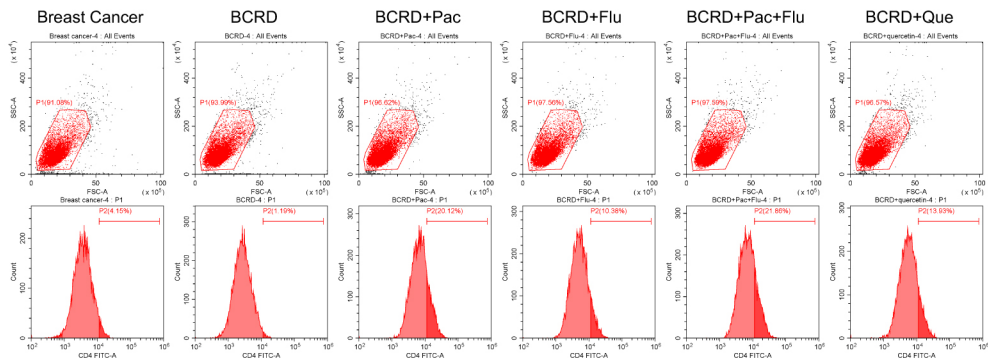**B****CD8+**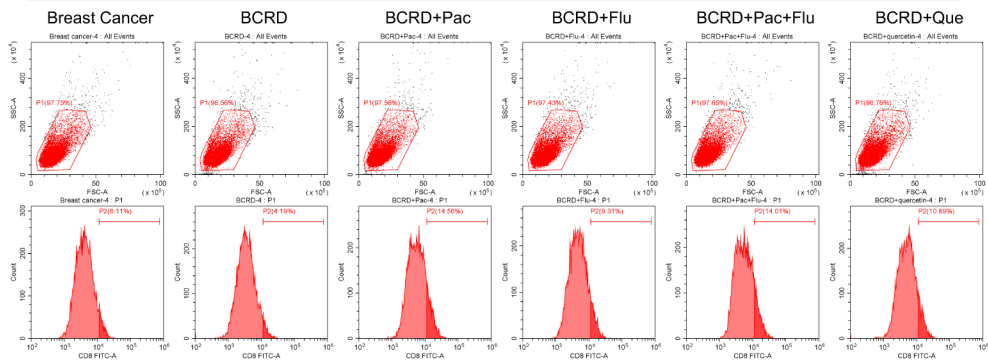

Supplement: Supplementary 3 — Supplementary Figure 2: CD4+ and CD8+ ratios in mice. [file 8011988.f3.pdf]

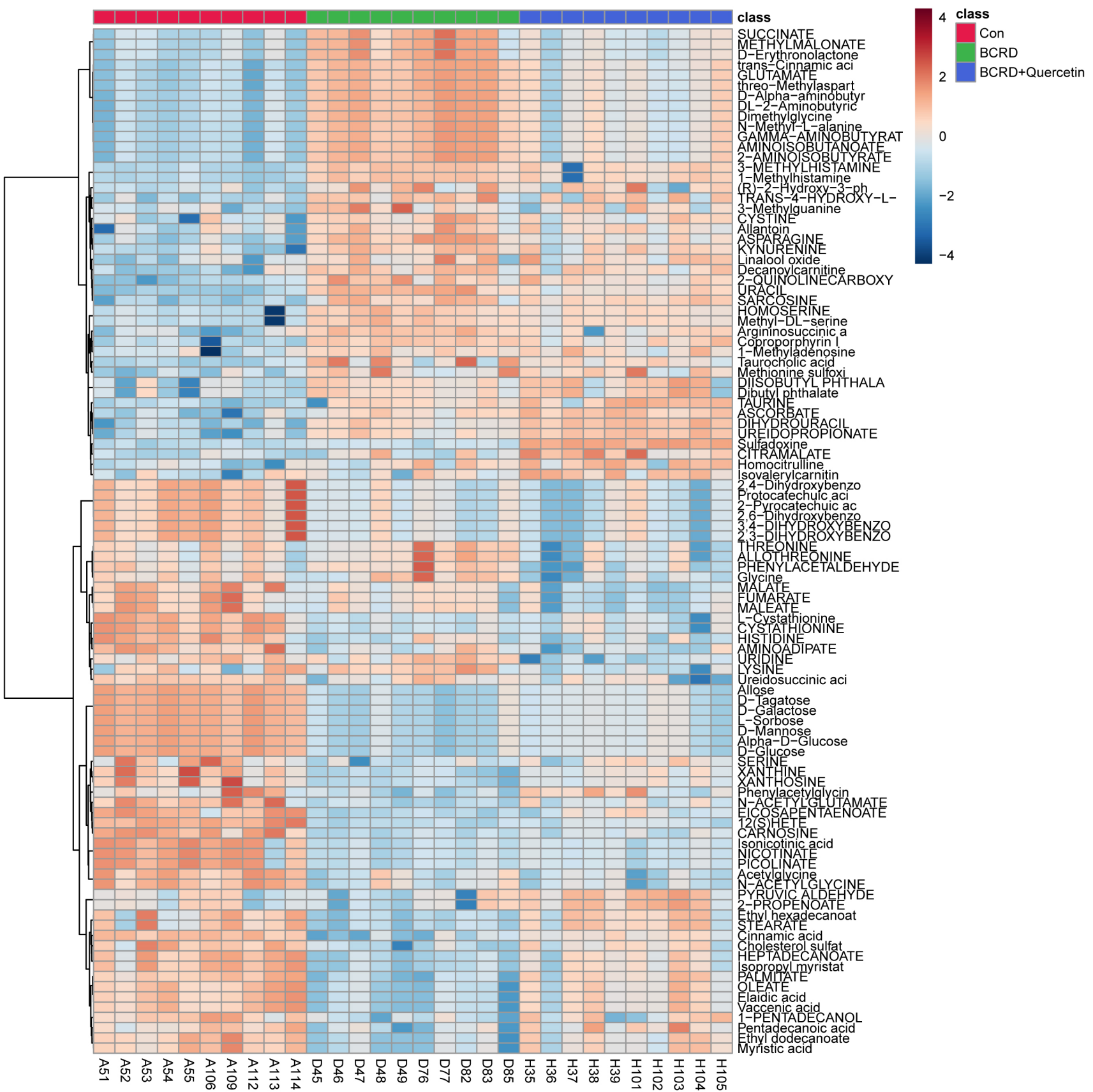

Supplement: Supplementary 4 — Supplementary Figure 3: the heat map of differential metabolites in plasma of mice. [file 8011988.f4.pdf]

**A**

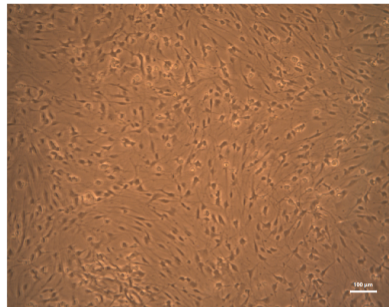

Primary Mouse Cortical Neurons

**B**

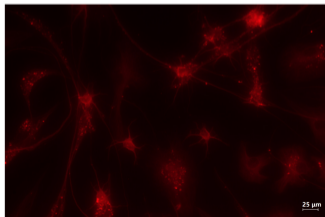

NSE

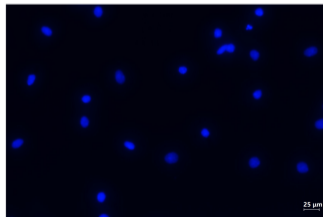

DAPI

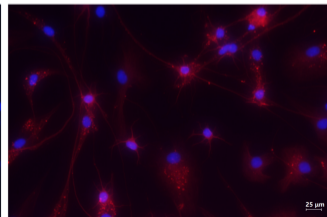

Merge

Supplement: Supplementary 5 — Supplementary Figure 4: primary mouse neuronal cell identification. (A) Representative image of primary mouse cortical neurons. (B) IF for identification of primary neurons. [file 8011988.f5.pdf]
